# Humoral Immune Response to Mixed *Pf*AMA1 Alleles; Multivalent *Pf*AMA1 Vaccines Induce Broad Specificity

**DOI:** 10.1371/journal.pone.0008110

**Published:** 2009-12-01

**Authors:** Kwadwo A. Kusi, Bart W. Faber, Alan W. Thomas, Edmond J. Remarque

**Affiliations:** 1 Department of Parasitology, Biomedical Primate Research Centre, Rijswijk, The Netherlands; 2 Department of Immunology, Noguchi Memorial Institute for Medical Research, College of Health Sciences, University of Ghana, Legon, Accra, Ghana; BMSI-A*STAR, Singapore

## Abstract

Apical Membrane Antigen 1 (AMA1), a merozoite protein essential for red cell invasion, is a candidate malaria vaccine component. Immune responses to AMA1 can protect in experimental animal models and antibodies isolated from AMA1-vaccinated or malaria-exposed humans can inhibit parasite multiplication *in vitro*. The parasite is haploid in the vertebrate host and the genome contains a single copy of AMA1, yet on a population basis a number of AMA1 molecular surface residues are polymorphic, a property thought to be primarily as a result of selective immune pressure. After immunisation with AMA1, antibodies more effectively inhibit strains carrying homologous *AMA1* genes, suggesting that polymorphism may compromise vaccine efficacy. Here, we analyse induction of broad strain inhibitory antibodies with a multi-allele *Plasmodium falciparum* AMA1 (*Pf*AMA1) vaccine, and determine the relative importance of cross-reactive and strain-specific IgG fractions by competition ELISA and *in vitro* parasite growth inhibition assays. Immunisation of rabbits with a *Pf*AMA1 allele mixture yielded an increased proportion of antibodies to epitopes common to all vaccine alleles, compared to single allele immunisation. Competition ELISA with the anti-*Pf*AMA1 antibody fraction that is cross-reactive between FVO and 3D7 AMA1 alleles showed that over 80% of these common antibodies were shared with other *Pf*AMA1 alleles. Furthermore, growth inhibition assays revealed that for any *Pf*AMA1 allele (FVO or 3D7), the cross-reactive fraction alone, on basis of weight, had the same functional capacity on homologous parasites as the total affinity-purified IgGs (cross-reactive+strain-specific). By contrast, the strain-specific IgG fraction of either *Pf*AMA1 allele showed slightly less inhibition of red cell invasion by homologous strains. Thus multi-allele immunisation relatively increases the levels of antibodies to common allele epitopes. This explains the broadened cross inhibition of diverse malaria parasites, and suggests multi-allele approaches warrant further clinical investigation.

## Introduction

Malaria continues to be one of the most important human parasitic diseases, with a global estimate of about 247 million clinical cases and almost 1 million deaths annually [1]. The greater burden of the disease is caused by *Plasmodium falciparum* in sub-Saharan Africa, where children under 5 years old, pregnant women (mostly primigravid) and their foetuses are at the greatest risk. A cost-effective vaccine would form a powerful additional component in control strategies for malaria and a number of *Plasmodium* antigens expressed at different stages of the parasite's complex life cycle are currently undergoing clinical evaluation [2].

Among the candidates in clinical testing is *Plasmodium falciparum* Apical Membrane Antigen 1 (*Pf*AMA1), a protein expressed in sporozoites and in merozoites of both liver and asexual erythrocytic development stages, the vaccine-related properties of which has recently been reviewed [3]. In brief, AMA1 is a merozoite membrane protein initially located in micronemes. Around the time of merozoite release from schizonts AMA1 is translocated to the merozoite surface, where it is involved in merozoite/red cell interactions preceding invasion [4]–[6]. Anti-AMA1 antibodies can interfere with AMA1 function and prevent invasion *in vitro* [7]–[10], this effect requiring immunisation with correctly folded AMA1 [11], [12]. The ectodomain of AMA1, which is the vaccine target, is shed as 44 and 48 kDa alternate proteins from the merozoite surface upon RBC invasion [13]. The amino acid sequence of the ectodomain has 16 cysteine residues that are conserved in all AMA1 sequences and these form disulphide bonds that result in a structure with three distinguishable but interactive domains (reviewed in [3]).

Polymorphism in AMA1 has long been evident [14], thought to be an effect of selection exerted by host immune responses [15], [16]. Immunisation with one allele of AMA1 ectodomain induces antibodies that inhibit homologous parasite growth *in vitro* to a greater extent as compared to heterologous parasites [12], [17], [18]. The induction of functional antibodies has been demonstrated in a number of ways, including rodent and primate challenge/passive immunisation studies [15], [19]–[21]. In some cases, and particularly with the rodent parasite *P. chabaudi*, antibodies are protective against parasites expressing homologous AMA1 but not those expressing heterologous AMA1 alleles [15], [22]. Antibodies to both conserved and strain-specific *Pf*AMA1 antibody epitopes have been observed in malaria-exposed humans [23], [24].

About 10% of amino acid residues of AMA1 are polymorphic, and even when appearing distant in the primary structure, may cluster within the tertiary structure [25]–[27]. These polymorphic clusters occur predominantly on one surface of the AMA1 molecule, which suggests that this face is accessible to antibody at the parasite surface [28]. This points to the significance of strain-specific epitopes in eliciting protective antibodies [12], [17], [29], although conserved AMA1 epitopes are also targets for inhibitory antibodies [30], [31].

Antibodies induced by immunisation with a combination of two allelic forms of *Pf*AMA1 (FVO and 3D7) inhibit the *in vitro* growth of both parasite strains to the same extent as antibodies raised against the single respective antigens [17], although there was no significant gain in growth inhibition against unrelated parasite strains. Similar observations from antigen recognition in ELISA have been reported in human trials with vaccine candidates incorporating *Pf*AMA1 proteins from the FVO and 3D7 parasite strains [32]–[34], and multi-allele immunisation has been proposed as one way of overcoming strain-specificity in *Pf*AMA1 responses. The question remains whether antibodies elicited by a multi-allele vaccine would be effective against parasites expressing a relatively distant natural *Pf*AMA1 allele from those of the vaccine components.

An effective *Pf*AMA1 vaccine will be required to overcome allelic diversity. We have therefore obtained sera from rabbits immunised separately with the full ectodomain *Pf*AMA1 from FVO, 3D7 and HB3 strains, as well as sera from rabbits immunised with a mixture of these 3 *Pf*AMA1 alleles, to assess the feasibility and mechanism of broadening the antibody response by immunisation with a mixture of *Pf*AMA1 alleles. Strain-specific and cross-reactive anti-*Pf*AMA1 antibody fractions were also used to assess the relative importance and contribution of these two IgG fractions to the overall *in vitro* functional capacity of anti-*Pf*AMA1 antibodies. The study was also used to validate the competition ELISA methodology for assessing antibody responses to naturally-occurring *Pf*AMA1 antigens and establish it as an analytical tool for dissecting humoral immune responses to polymorphic antigens. Our data shows the feasibility of broadening the functional antibody response to *Pf*AMA1 by immunisation with a mixture of three *Pf*AMA1 alleles. Such a vaccine preferentially induces the expression of antibodies to epitopes that are common to the vaccine component alleles by diluting out responses to the strain-specific epitopes. Common or cross-reactive antibodies, in the absence of strain-specific antibodies, are capable of inhibiting the *in vitro* growth of parasites represented by the vaccine *Pf*AMA1 alleles as well as parasites expressing *Pf*AMA1 alleles not included in the vaccine. The data indicates that *Pf*AMA1 multi-allele vaccine strategies should be pursued and provide a justification for further clinical investigation.

## Methods

### Protein Production and Rabbit Immunisations

The full ectodomain of AMA1 allelic forms from the *P. falciparum* strains FVO, HB3, 3D7 and CAMP were expressed in *Pichia pastoris* by a similar methodology as described elsewhere [35], [36]. Potential N-glycosylation sites were removed from the *Pf*AMA1 gene sequences by mutagenesis before expression.

All animals were handled in strict accordance with good animal practice as defined by the Belgian national animal welfare regulations, and all animal work was approved by the ethics committee of the Centre d'Economie Rurale (CER Groupe, Marloie, Belgium). Rabbit immunisations were done intramuscularly (Eurogentec SA, Seraing, Belgium) with 4 doses of *Pf*AMA1 formulated in Montanide ISA720 (Seppic, Paris, France) as adjuvant, according to the manufacturer's instructions. New Zealand white rabbits were immunised on days 0, 28, 56 and 82, and the final bleed sera collected on day 95 were used in this study. Four groups of 2 rabbits each were immunised as follows: the first 3 groups received 30 µg/dose of FVO AMA1, 3D7 AMA1 or HB3 AMA1 respectively. The 4^th^ group received 30 µg/dose of a mixture (10 ug each) of the 3 *Pf*AMA1 alleles, also formulated in the same adjuvant.

### Antibody Purification

Serum antibodies were purified on Protein A sepharose (GE Healthcare, Etten-Leur, The Netherlands) columns. Binding and elution buffers (Pierce, Rockford, IL) were used according to manufacturer's protocols. After elution, antibodies were concentrated and exchanged into RPMI 1640 using AmicronUltra-15 tubes (30-kDa cutoff; Millipore, Ireland). Antibodies were subsequently sterile-filtered with 0.22 µm Ultrafree MC centrifugal filter units (Millipore), the concentration determined with a Nanodrop ND1000 spectrophotometer (Nanodrop Technologies, Wilmington, DE) and stored at −20°C until use.

3D7-specific AMA1 antibodies were purified from the total serum IgGs (protein A purified) of a 3D7-immunised rabbit using a 3D7 AMA1-coupled sepharose matrix. The eluted 3D7-specific IgG fraction was further separated into strain-specific (flow-through) and cross-reactive (eluate) IgGs by passage over an FVO AMA-coupled sepharose matrix. Protein A purified anti-FVO AMA1 antibodies were also affinity fractionated as has been described for anti-3D7 AMA1, first over an FVO AMA1-coupled sepharose matrix, then the eluate over a 3D7 AMA1-coupled matrix (Figure S1). The constituent IgG in all fractions were confirmed by ELISA and all fractions were concentrated, sterile filtered and stored at −20°C until use.

### ELISA

Competition ELISA assays were carried out with AMA1 from 4 different *P. falciparum* strains (FVO, HB3, 3D7 and CAMP) to define specificities of antibodies raised against the 3 *Pf*AMA1 vaccine antigens (FVO, 3D7, HB3). An initial titration was performed to determine the optimal dilution of purified IgG required for the competition assay with each of the 3 vaccine antigens as coating antigen. Briefly, 96-well flat bottom Microlon titre plates (Greiner, Alphen a/d Rijn, The Netherlands) were coated with 100 µl/well of 1 µg/ml FVO, 3D7 or HB3 AMA1 ectodomain at 4°C overnight. Plates were washed 5 times with PBS, 0.05% Tween 20 (PBS-T) using an automated plate washer (Bio-TEK Instruments, Inc, VT) and blocked with 200 µl/well of 3% BSA in PBS-T for at least 1.5 h. IgG samples were titrated 3-fold from 1∶10,000 and incubated for 2 h. A pool of sera from rabbits immunised with a mixture of the 3 *Pf*AMA1 alleles, titrated 2-fold from 1∶100,000, was used as standard calibrator on all plates. Protein A purified IgG from rabbit pre-immunisation sera, titrated 2-fold from 1∶10,000, was used as negative control on all plates. All samples, standards and controls were diluted with 0.5% BSA in PBS-T and added in duplicate (100 µl/well). After sample incubation, plates were washed and 100 µl/well of 1∶1250-diluted goat anti-rabbit IgG/alkaline phosphatase conjugate (Pierce, Rockford, IL) added for 1 h. Plates were then washed, incubated with 100 µl/well p-nitrophenyl phosphate (pNPP; Fluka, Poole, UK) as substrate for 30 min. and the optical density (OD) at 405 nm read with a 96-well ELISA plate reader (BioRad, Japan).

ODs were converted to arbitrary units (AUs) by the standard curve included on each plate using an excel-based four-parameter logistic function, which after correcting for variation approximates the IgG dilution that gives an OD of 1.0 to one arbitrary unit (1 AU).

Dilutions that resulted in an AU of 2 were extrapolated for each rabbit serum/purified IgG and used for the subsequent antigen competition assay. The assay involved co-incubation of different allelic forms of *Pf*AMA1 with the same dilution of test IgGs in plates coated entirely with one of the vaccine *Pf*AMA1 alleles, such that there was competition between the added (competitor) antigens and the coated antigen for binding to test IgGs. The procedure was similar to that described for the pre-titration, except for the addition of 50 µl/well rabbit IgG at 2 times the desired dilution (equivalent to an AU of 4) to 50 µl/well of titrated soluble antigens in coated and blocked plates. Antibodies from all rabbits were co-incubated with the 4 *Pf*AMA1 alleles (FVO, HB3, 3D7, CAMP) separately, on plates coated with either FVO, HB3 or 3D7 AMA1. The competitor/soluble antigens were titrated 3-fold from 30–0.005 µg/ml over 9 duplicate wells, and the 10th sample wells were left without soluble antigen. The appropriately diluted IgG sample was then added to all 10 duplicate wells for each soluble antigen and after incubation for 2 h, plates were developed as described above.

To further dissect the nature and underlying mechanism of humoral responses to the different *Pf*AMA1 alleles, IgG pools made from the single allele immunisations, were also compared with IgGs from the single and multi-allele immunisations by competition ELISA.

Duplicate OD values (from residual antibody binding to the coated antigen after competition) for wells that had soluble antigens were converted to arbitrary units and expressed as a percentage of AU values from wells without soluble antigen. The percent residual binding values were then plotted (points) alongside the predicted percent values (curves) based on a least squares approximation from the following four-parameter logistic function;
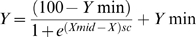
where Y is the predicted % residual binding, ***Y_min_*** is the maximal depletion at infinite soluble antigen concentration (minimum value), ***X*** is the soluble antigen concentration (log scale), ***X_mid_*** is the soluble antigen concentration (log scale) at which 50% antibody depletion is achieved (midpoint between the maximum and minimum depletion values), and ***sc*** is the slope of the curve. Percent antibody depletion for any competitor/soluble antigen is therefore the difference between 100% (binding in the absence of soluble antigen) and the residual binding.

The competition assay was initially validated by testing anti-FVO AMA1 IgG or serum at dilutions equivalent to 0.2, 0.5, 1, 2, 4 and 8 times the titre (1 AU) on FVO-coated plates (100 ng/well) with the same soluble antigen concentrations (3-fold titration from 30 µg/ml over 9 duplicate wells). The assay was shown to be reproducible and independent of the antibody source (serum or purified IgG) and the dilution provided the OD values in wells with no competitor antigen were within the linear portion (ODs of 0.3–2.5 over blank) of the standard curve.

### Antibody Avidity Measurements

The binding capacity of antibodies raised by single and mixed allele immunisations were determined by avidity ELISA with sodium isothiocyanate (NaSCN) elution. Briefly, 96-well flat bottom Microlon titre plates were coated with AMA1 allelic antigens as described above, and after blocking, incubated with a pre-determined titre (1 AU) of sera from immunised rabbits for 1 h. Plates were then washed and incubated with an increasing concentration of NaSCN (0, 0.25, 0.5, 1.0, 1.25, 1.5, 1.75, 2.0, 2.25, 2.5 and 3.0 M) in different duplcate wells for 15 min. Plates were again washed and subsequently developed with goat anti-rabbit IgG/alkaline phophatase conjugate and pNPP substrate as already described. Avidity index, the concentration of NaSCN required for 50% dissociation of bound antibodies (relative to duplicate wells without NaSCN) was the extrapolated in Microsoft excel for each rabbit serum sample.

### Parasite Cultures and Growth Inhibition Assays

Protein A and affinity-purified IgG fractions were tested for *in vitro* activity in parasite growth inhibition assays (GIAs) as described elsewhere [18]. All IgGs and IgG pools were tested in triplicate on FCR3, NF54, HB3 or CAMP parasite strains at a 3-fold serial dilution from 6 mg/ml (protein A purified IgG) or 1 mg/ml (affinity-purified IgG) in 96-well culture plates. Parasites were cultured under standard conditions (an atmosphere of 5% CO_2_, 5% O_2_, and 90% N_2_, 37°C), and the *Pf*AMA1 antigen expressed by all parasite strains were verified by PCR and restriction fragment length analysis. Parasite cultures were mycoplasma-free and synchronized with 0.3 M alanine, 10 mM Hepes pH 7.5 before use in an assay. Late trophozoite/early schizont stages at a parasitaemia of 0.3±0.1% and 2% final haematocrit were used in all assays. The final culture volume was 50 µl/well and parasites were incubated for 40–45 hrs. Parasite growth was assessed by measuring parasite lactate dehydrogenase levels with the lactate/diaphorase/APAD substrate system, and plates were read at 655 nm after 30 min of development. Parasite growth inhibition was expressed as;
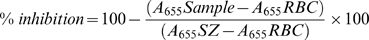
where *A_655_Sample* is the OD_655_ for any test sample well, *A_655_S*Z is the average OD_655_ of schizont control wells included on each plate and *A_655_RBC* is the average OD_655_ of RBC control wells. The data was presented as the arithmetic mean % inhibition from each sample triplicate.

### Statistical Analyses

Residual binding (or minimum) values in competition ELISA, and the corresponding confidence intervals were generated by a 4-parameter logistic fit with least squares approximation using the *R* statistical package (R Development Core Team, 2008, version 2.8.1). Comparisons between minimum values estimated in the non-linear regression were done with Student's *t* test; *p* values<0.05 were considered statistically significant. All plots were prepared with the *R* statistical package. Since antibody depletion patterns in ELISA were similar for IgG samples from the two rabbits in each immunisation group, data presented (ELISA and GIA) are for only one rabbit per group and is therefore of a qualitative nature.

## Results

### Competition ELISA Validation

The competition ELISA assay used was based on coating plates with one allele of *Pf*AMA1 and mixing test antibody samples with the same or other *Pf*AMA1 alleles to determine the degree to which antibody binding to the coating material was inhibited. The assay was reproducible within and between runs and at various initial serum/IgG concentrations (CV was generally below 2%, and maximally 15% for very low OD values). Antibody depletion patterns were similar irrespective of whether serum or protein A-purified IgG samples were used for the assay (data not shown). Depletion patterns, plotted as percentages, were similar irrespective of the final serum/IgG dilution used provided the OD value was within the linear portion of the standard curve. An 8-fold active dilution range (0.5–4 AU) gave optimal results (Figure S2). Dilutions above this range (8 AU or higher) shifted the curves to the left (suggesting less antigen required for depletion compared to dilutions within the linear range), and dilutions below the range (0.2 AU or lower) shifted curves to the right (suggesting more antigen required for depletion compared to dilutions within the linear range) (Figure S2). Antibody depletion patterns were also very similar for each pair of rabbits immunised with the same antigen, irrespective of the original antibody response/titre (data not shown).

A 4-parameter logistic plot was used to assess the reproducibility and robustness of the assay by comparing data from three different assays and at three different antibody dilutions using final ODs within the linear portion. A statistical comparison of the minimum values, the most informative parameter for comparing antibody specificities by this assay, is presented in Table 1 for an assay involving co-incubation of 4 different competitor antigens with total IgG from an FVO AMA1 immunisation in FVO AMA1-coated plates. For any competitor antigen, the minimum value represents the proportion of antibodies that do not bind to the competitor antigen, but do bind to the coating antigen. This fraction of the test antibodies represents the strain-specific component with respect to the competitor antigen, while the fraction depleted by the competitor antigen represents antibodies that are capable of binding to both the coated and competitor antigens. With the exception of HB3 AMA1, competitor antigens had highly consistent minimum values between assays and with different starting antibody dilutions. Minimum values for 3D7 and CAMP AMA1 competitor antigens were consistently and significantly different from the homologous FVO AMA1 competitor antigen. The shape of the HB3 AMA1 competitor antigen curve suggested that at higher concentrations, further antibody depletion was possible. This was not the case for the other competitor antigens as the minimum values had almost reached a plateau at the highest antigen concentration used. As a result, relatively wide confidence intervals were obtained for minimum values when HB3 AMA1 competed for anti-FVO AMA1 antibodies (Table 1).

**Table 1:** Residual IgG binding estimates from competition ELISA validation.

	Competitor antigen (*Pf*AMA1)			
	FVO	HB3	3D7	CAMP
Expt.1 (1 AU)	4.2 (−1.8–10.2)	14.2 (−3.8–32.2)	50.1 (43.7–56.5)	33.1 (29.3–36.8)
Expt.1 (2 AU)	3.3 (−1.9–8.5)	8.4 (−18.3–35.0)	50.2 (45.0–55.3)	34.1 (29.6–38.5)
Expt.2 (2 AU)	3.7 (−1.3–8.6)	22.4 (14.8–29.9)	49.9 (42.0–57.9)	31.6 (25.5–37.6)
Expt.3 (4 AU)	6.6 (1.2–12.0)	5.4 (0.1–10.6)	51.6 (42.6–60.7)	36.2 (28.0–44.4)
Mean of all Expts.	4.4 (0.3–8.6)	13.5 (6.1–20.9)	50.4 (44.9–55.9)	33.7 (29.8–37.5)

The assay was validated by repeated assessment of anti-FVO AMA1 antibody depletion by FVO, HB3, 3D7 and CAMP AMA1 competitor/soluble antigens, on FVO AMA1-coated plates. Values are the estimated minimum residual binding and reported as minimum value (95% CI) for each of the competitor/soluble antigens. Assays were performed on three different days with three different antibody dilutions (1 AU, 2 AU, 4 AU). 1 AU is equivalent to the antibody titre.

The slope describes an inverse relationship between antibodies not depleted by the competitor antigen and the log-concentration of competitor antigen, with a higher absolute value representing a steeper curve. At different antibody dilutions for FVO, 3D7 and CAMP AMA1 competitor antigens, the slope was fairly consistent between assays (−0.26 to −0.52), with most values around −0.40. *Xmid*, like IC_50_, is the concentration of competitor antigen that results in 50% antibody depletion. However, interpretation of this parameter is confounded by the different minimum values for the different competitor antigens (Figure S2).

### Antibodies from Multi-Allele Immunisation Have an Increased Cross-Reactivity

**Figure 1:**
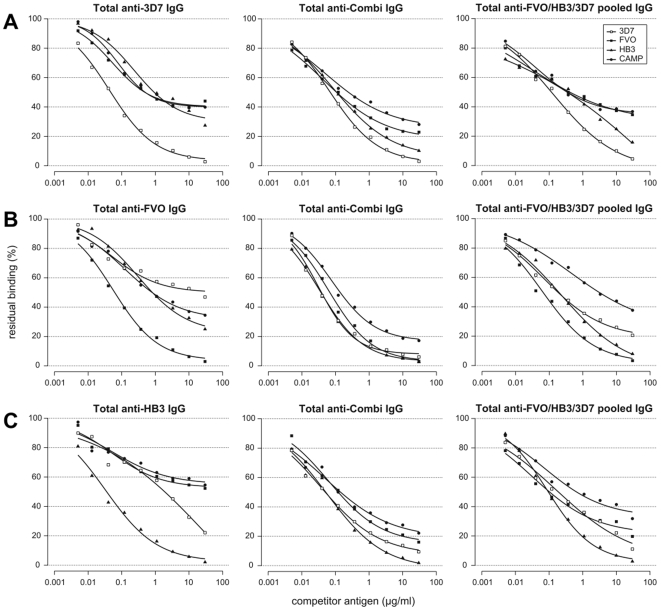
Competition ELISA with protein A-purified antibodies and antibody pools made from single allele immunisations. A) Assay on 3D7 AMA1-coated plates with anti-3D7 AMA1, anti-Combi (mixed allele immunisation) and anti-FVO/HB3/3D7 antibody pool. B) Assay on FVO AMA1-coated plates with anti- FVO AMA1, anti-Combi and anti-FVO/HB3/3D7 antibody pool. C) Assay on HB3 AMA1-coated plates with anti- HB3 AMA1, anti-Combi and anti-FVO/HB3/3D7 antibody pool. IgG pools were made from antibodies raised in single allele immunisations with FVO, HB3 and 3D7 AMA1. All assays were performed with FVO, HB3, 3D7 and CAMP AMA1 proteins as competitor antigens. All IgGs were used at 2 times the pre-determined antibody titre. Plots are representative of at least 2 assay repeats using IgGs from one rabbit per group since the depletion patterns were similar for both rabbits in each immunisation group.

Once validated, the competition ELISA assay was used to compare the relative proportions of cross-reactive and strain-specific antibody fractions induced by single and mixed *Pf*AMA1 allele immunisations. Antibody depletion patterns revealed significantly increased cross recognition of all heterologous competitor antigens by antibodies from the mixed allele immunisation (anti-Combi) compared to those from the single allele immunisations (Figure 1, Table 2). This implies that there is a higher proportion of antibodies to common epitopes in the mixed allele immunisation compared to the proportion in any single allele immunisation. For example, recognition and depletion of antibodies by the FVO AMA1 competitor antigen increased significantly from 60% (40.2% residual binding) for IgGs from the 3D7 AMA1 single allele immunisation to over 80% (17.8% residual binding) for anti-Combi antibodies (p<0.0001) in competition assays with 3D7 AMA1 as coating antigen (Figure 1A). Similarly, depletion by HB3 AMA1 in the same assays was almost 70% (29.9% residual binding) with anti-3D7 AMA1 antibodies and up to 95% (5.73% residual binding) with anti-Combi antibodies (p = 0.0006). Depletion by CAMP AMA1, which was not a component antigen of the mixed allele vaccine, also increased significantly from 60% (39.9% residual binding) with anti-3D7 AMA1 antibodies to almost 75% (25.7% residual binding) with anti-Combi antibodies (p = 0.0008). Similar trends of increasing recognition and depletion were observed by comparing antibodies from single allele immunisation with HB3 AMA1 and FVO AMA1 with the mixed allele immunisation when assays were performed with the respective *Pf*AMA1 alleles as coating antigens (Figures 1B and 1C). Depletion of anti-Combi antibodies was consistently highest for all competitor antigens when FVO AMA1 was used as the coating antigen. CAMP AMA1 highly recognized and depleted anti-FVO AMA1 antibodies (31.6% residual binding) and least recognized anti-HB3 antibodies (55.0% residual binding), but relatively similar degrees of anti-Combi antibodies were recognized/depleted by CAMP AMA1 irrespective of the coating antigen used (Table 2).

**Table 2:** Residual IgG binding estimates for antibodies raised in single and multi-allele immunisations.

Coating antigen	IgG Sample		Competitor antigen (*Pf*AMA1)		
		**FVO**	**HB3**	**3D7**	**CAMP**
	anti-FVO	3.7 (−1.3–7.2)	22.4 (14.8–29.9)	49.9 (42.0–57.9)	31.6 (25.9–37.2)
FVO AMA1	anti-Combi	3.0 (−1.3–7.2)	2.9 (0.1–5.8)	8.0 (3.1–12.9)	16.8 (12.6–20.9)
	IgG pool§	1.9 (−7.1–10.9)	−3.2* (−15.9–9.4)	18.6 (12.5–24.7)	25.5 (14.1–36.8)
		**FVO**	**HB3**	**3D7**	**CAMP**
	anti-3D7	40.2 (36.8–43.6)	29.9 (19.6–40.2)	3.3 (−0.8–7.4)	40.0 (36.0–43.8)
3D7 AMA1	anti-Combi	17.8 (13.4–22.2)	5.7 (2.4–9.0)	1.9 (−1.5–5.4)	25.7 (19.2–32.2)
	IgG pool§	29.9 (18.8–40.9)	−150* (−546–246)	−3.0* (−11.1–5.0)	34.6 (28.9–40.3)
		**FVO**	**HB3**	**3D7**	**CAMP**
	anti-HB3	52.7 (46.2–59.1)	2.3 (−6.0–10.5)	−57.7* (−299–184)	55.0 (43.0–67.0)
HB3 AMA1	anti-Combi	15.1 (7.6–22.5)	−2.2* (−11.7–7.4)	7.5 (1.5–13.5)	18.8 (8.2–29.5)
	IgG pool§	21.2 (12.6–29.8)	2.1 (−2.3–6.5)	3.3 (−16.8–23.4)	32.9 (22.1–43.7)

Residual binding values are the predicted minimum values based on the measured values for each competitor antigen, and were generated with a four-parameter logistic fit with least squares approximation. Values are estimated with the R statistical package and reported as % residual binding or minimum value (95%CI).

§ A pool of IgGs from the single allele immunisations with FVO, HB3 and 3D7 AMA1.

* Negative estimate of residual binding (minimum values have not reached a plateau yet). Minimum values cannot be accurately estimated.

To further assess the nature of antibodies raised in a mixed allele immunisation, protein A-purified antibody pools containing anti-FVO/HB3, anti-FVO/3D7 and anti-HB3/3D7, as well as a pool of antibodies against all three *Pf*AMA1 alleles (anti-FVO/HB3/3D7) were made from antibodies raised in the single allele-immunised rabbits. These antibody pools were compared to antibodies from single and mixed allele immunisations in competition ELISA with FVO, HB3 or 3D7 AMA1 as coating antigens. Depending on the coating antigen/IgG pool combination, there were small changes in depletion by the four native *Pf*AMA1 alleles compared to the IgGs from single allele immunisations, with mixed significance (data not shown). The antibody pools with anti-HB3 IgGs were predictably less recognized by CAMP AMA1. Recognition/depletion patterns of the pool of three (anti-FVO/HB3/3D7) were intermediate between the observed patterns for antibodies from single and mixed allele immunisations (Figure 1). Generally, there was the tendency for greater depletion (lower residual binding) of anti-Combi antibodies by all competitor antigens compared to the depletion of antibodies from the anti-FVO/HB3/3D7 IgG pool (Table 2). This suggests that although IgG pooling would result in a decreased proportion of strain-specific antibodies, immunisation with the mixed antigens yields even lower levels of antibodies to epitopes that are specific to the component vaccine antigens.

**Figure 2:**
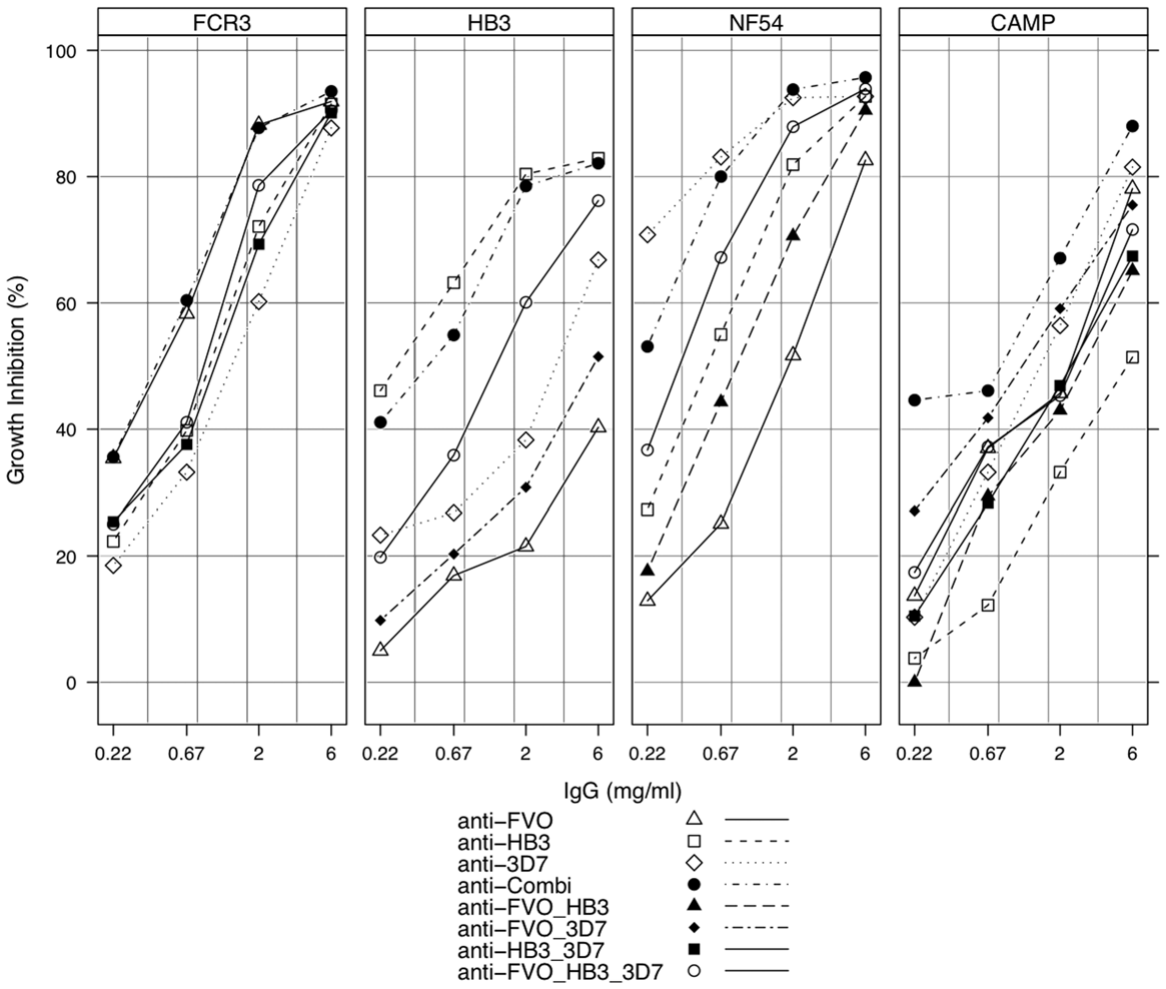
Growth inhibition levels exhibited by protein A-purified IgGs from single/mixed *Pf*AMA1 immunisations and IgG pools. All IgG fractions were tested in a single growth cycle assay with FCR3, HB3, NF54 and CAMP strains of *P. falciparum*. For all strains, assays were performed with 0.3±0.1% parasitaemia and a final haematocrit of 2%. IgG samples were tested at 4 dilutions (3-fold titration from 6 mg/ml). IgG pools were made from antibodies raised in single allele immunisations with FVO, HB3 and 3D7 AMA1. The data presented is representative of at least two assay repeats using IgGs from one of the two rabbits per group.

In order to assess the functional capacity of these antibodies in relation to the observations made by competition ELISA, growth inhibition assays were performed with the FCR3 (one pro-sequence amino acid difference from FVO AMA1), HB3, NF54 (with identical AMA1 to clone 3D7) and CAMP strains of *P. falciparum*. In assays with NF54, HB3 and FCR3 parasite strains, growth inhibition levels decreased more rapidly with decreasing concentration of antibodies against heterologous *Pf*AMA1 alleles (Figure 2). Thus the extent of *in vitro* growth inhibition of any parasite strain was dependent on the antibody source (homologous versus heterologous), and generally for heterologous parasites, also on the number of amino acid variants between the vaccine and parasite AMA1 alleles (see Table 3). For example, the growth of NF54 parasites was best inhibited by anti-3D7 AMA1 antibodies and least by anti-FVO AMA1 antibodies over the 4 dilutions tested, with anti-HB3 AMA1 antibodies yielding intermediate inhibition (Figure 2). Most importantly, however, anti-Combi antibodies resulted in growth inhibitions that were comparable with that of antibodies from single allele immunisations on the respective homologous parasite strains in all cases. Furthermore, the anti-Combi antibodies yielded the best growth inhibition of CAMP parasites compared to antibodies from the 3 single allele immunisations, even though CAMP AMA1 was not included in the mixed allele vaccine. The relatively similar ELISA titres of mono-specific antibodies (950,000 for anti-FVO, 1,150,000 for anti-HB3 and 1,280,000 for anti-3D7 AMA1) on the respective homologous *Pf*AMA1 alleles compared to anti-Combi antibodies (1,230,000 on FVO, 1,140,000 on HB3 and 1,120,000 on 3D7 AMA1) as measured in protein G-purified fractions eliminates the possibility of this observation being due to higher titres of anti-Combi antibodies. The observed GIA activity cannot also be attributed to a better quality of the anti-Combi antibodies since these were shown to have antigen-binding capacities in the same order as mono-specific antibodies when titres were normalized (Table 4). The current observation may therefore be mainly attributed to the induction of a more cross-reactive antibody profile and represents a broadened inhibitory capacity of anti-Combi antibodies compared to antibodies induced in single allele immunisations.

**Table 3:** Number of amino acid variants between vaccine and *Pf* parasite AMA1 alleles.

Vaccine antigen	Parasite strain			
	NF54	HB3	FCR3	CAMP
3D7	* **6** (2,3,1)	**29** (15,8,6)	**30** (19,8,3)	**26** (14,9,3)
HB3	**30** (16,8,6)	* **5** (1,3,1)	**24** (13,7,4)	**31** (17,8,6)
FVO	**30** (19,8,3)	**23** (12,7,4)	* **6** (2,3,1)	**20** (11,6,3)

Values represent only the differences in domains I, II and III of *Pf*AMA1 ectodomain. Differences per domain have been presented in brackets as (domain I, domain II, domain III).

* The 6 variant amino acids between “homologous” AMA1 alleles (5 for HB3) are due to amino acid substitutions introduced to prevent protein glycosylation and cleavage. Substitutions occur at positions 162 and 288 in domain I (position 288 only for HB3), positions 373, 422 and 423 in domain II, and position 499 in domain III.

**Table 4:** Measured avidity Indices for mono-specific and anti-Combi antibodies against vaccine *Pf*AMA1 alleles.

	Avidity Index by capture antigen		
Rb ID (Immunising Ag)	FVO	HB3	3D7
1455 (FVO)	1.23	0.91	1.01
1456 (HB3)	0.89	1.10	0.81
1459 (3D7)	1.15	1.03	1.38
1461 (Combi)	1.04	1.06	1.31

Avidity index of AMA1-specific antibodies was estimated as the concentration of NaSCN required to dissociate 50% of AMA1-bound antibodies. The avidity indices of mono-specific antibodies were determined against both the immunising (“homologous”) allele and the other two “heterologous” alleles. That for anti-Combi antibodies was determined against all vaccine component alleles.

GIAs with the antibody pools were also performed to assess the trends in growth inhibition with respect to the varying antibody specificities. Results showed that differences in growth inhibition between the pools were dependent on the parasite strain/antibody pool combination. The anti-FVO/3D7 antibody pool, for example, inhibited the growth of CAMP parasites better (Figure 2) than the other double pools (anti-FVO/HB3, anti-HB3/3D7), even though these pools had similar antibody titres as measured by ELISA (data not shown). This is likely due to the fact that HB3 AMA1, being the most distant allele from CAMP AMA1 in terms of amino acid residues, shares fewer functional epitopes with CAMP AMA1 such that the pools with anti-HB3 AMA1 antibodies cross-reacted least with CAMP AMA1, resulting in relatively lower parasite growth inhibitions.

Inhibition of all strains by anti-Combi antibodies was higher compared with the anti-FVO/HB3/3D7 IgG pool, confirming the induction of higher levels of antibodies to common epitopes by multi-allele immunisation. Antibodies to common allele epitopes are predominantly induced since strain-specific epitopes on each of the vaccine alleles have been diluted out in the vaccine antigen mixture, and this translates to a broadened scope of *Pf*AMA1 recognition. The functional assay therefore confirms observations made by competition ELISA, and shows that mixed allele immunisation predominantly yields antibodies to common allele epitopes and low levels of antibodies to strain-specific epitopes. High levels of antibodies to the common vaccine allele epitopes are invariably required for broad strain inhibition.

### Most Cross-Reactive Antibody Epitopes Are Shared by All Alleles

**Figure 3:**
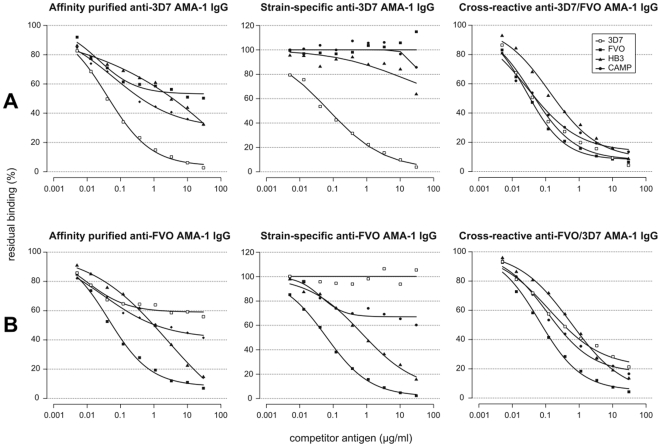
Competition ELISA with *Pf*AMA1-specific IgG fractions. Anti-3D7 AMA1 IgGs were affinity-purified from total (Protein A) IgG of one of the 3D7 AMA1-immunised rabbits. A portion of this IgG fraction was afterwards fractionated into 3D7 AMA1 strain-specific IgG (flow through) and 3D7/FVO cross-reactive IgG (eluate) by passage over an FVO AMA1 affinity matrix. Similar specific fractions were made from total IgGs from one of the FVO AMA1-immunised rabbits, first over an FVO AMA1 matrix, and then over a 3D7 AMA1 matrix. All IgG fractions were used for competition assays at 2 times the pre-determined antibody titre. AMA1 antigens from the 3D7, HB3, FVO and CAMP parasite strains were used as competitor antigens in all assays. Assays were done using plates coated with 3D7 AMA1 (A) and FVO AMA1 (B), and plots are representative of data from at least 2 repeat assays.

To assess the relative contributions of strain-specific and cross-reactive antibodies to overall antigen recognition and parasite inhibition, strain-specific and cross-reactive antibody fractions were affinity-purified from total IgGs of rabbits immunised with 3D7 AMA1 alone and FVO AMA1 alone. Cross-reactive and strain-specific IgG fractions for each of the two *Pf*AMA1 alleles were prepared with respect to the other allele (procedure presented schematically in Figure S1). Up to 90% of recovered IgGs from anti-3D7 AMA1 antibodies were cross-reactive with FVO AMA1, while over 95% of recovered IgGs from anti-FVO AMA1 antibodies were cross-reactive with 3D7 AMA1. These IgG fractions were compared with the respective un-fractionated affinity-purified anti-3D7 or anti-FVO IgGs by competition ELISA. The strain-specific fraction of anti-3D7 AMA1 IgGs had very little reactivity with HB3 and CAMP AMA1 alleles (Figure 3A). There was however, an improved recognition and depletion of IgGs in the cross-reactive fraction by all the heterologous *Pf*AMA1 alleles used (FVO, HB3, CAMP). Similar observations were made with anti-FVO AMA1 strain-specific and cross-reactive IgG fractions, except that the strain-specific fraction of anti-FVO AMA1 IgG was still highly reactive with HB3 AMA1, and to a lesser extent with CAMP AMA1 (Figure 3B). Based on these observations, it is likely that FVO AMA1 may induce the production of antibodies that are more cross-reactive in comparison with 3D7 AMA1.

**Figure 4:**
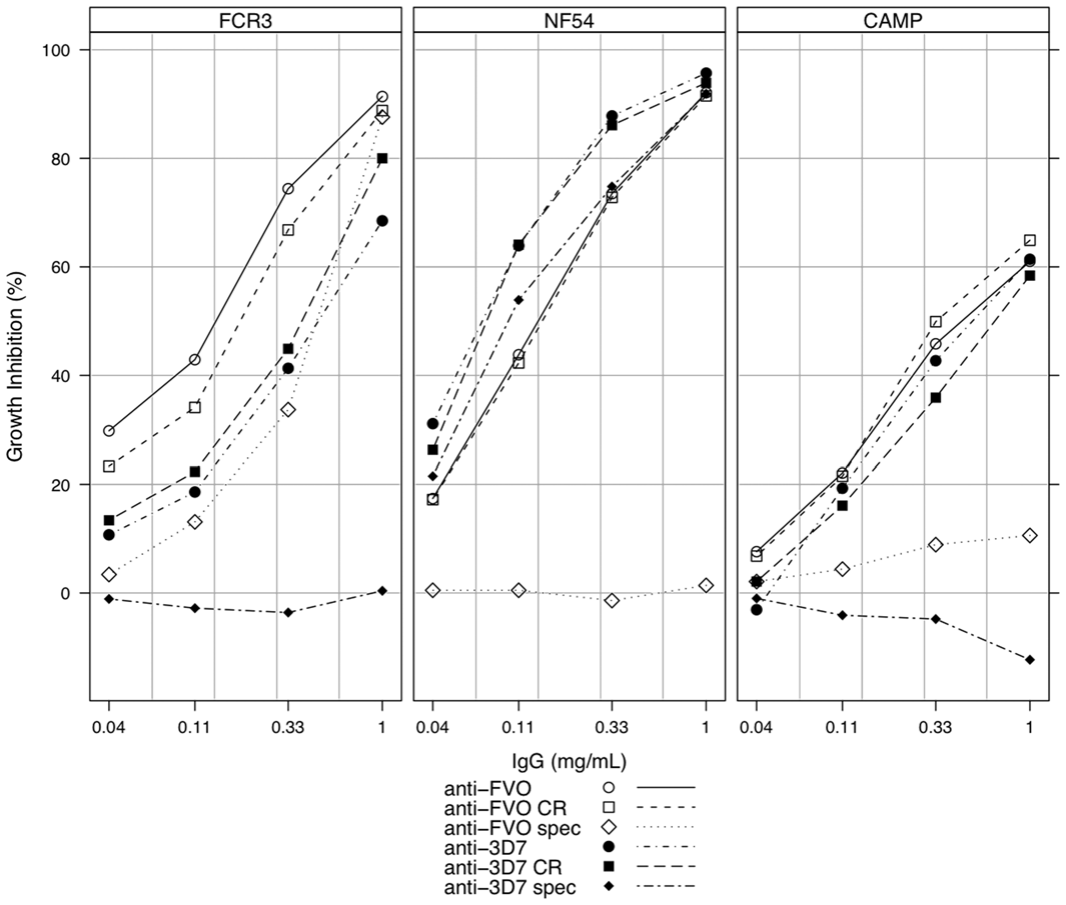
Representative data showing levels of parasite growth inhibition exhibited by affinity-purified AMA1-specific IgG fractions. All fractions were tested for functional activity on FCR3, NF54 and CAMP strains of *P. falciparum*. For all strains, assays were done with 0.3±0.1% parasitaemia and a final haematocrit of 2%. IgG samples were tested at 4 dilutions (3-fold titration from 1 mg/ml). *Anti-FVO* and *anti-3D7* are the respective total affinity-purified IgGs, *anti-FVO CR* and *anti-3D7 CR* designate the cross-reactive fractions, while *anti-FVO spec* and *anti-3D7 spec* designate the respective strain-specific fractions.

The affinity-purified antibody fractions were also tested for functional capacity by *in vitro* growth inhibition assays on FCR3 (FVO), CAMP and NF54 (3D7) parasite strains (Figure 4). The cross-reactive fractions alone had the same functional capacity on homologous parasites as the respective total affinity-purified IgGs when both were tested at the same concentrations. The anti-3D7 cross-reactive fraction showed slightly less inhibition on FCR3 heterologous parasites over the four antibody concentrations tested as compared to the anti-FVO cross-reactive fraction, and the reverse was true for the inhibition of NF54 parasites. Furthermore, both cross-reactive fractions yielded slightly lower inhibition of CAMP parasites compared to the inhibitions observed for same fractions on their respective homologous parasites. By contrast, the strain-specific fractions showed slightly less inhibition of red cell invasion by homologous parasites compared to the cross-reactive and total fractions. Both strain-specific fractions had negligible inhibitory effect on heterologous parasites, including the CAMP heterologous strains. These observations confirm the need to induce cross-reactive antibodies in overcoming allelic diversity to *Pf*AMA1, but also show that the cross-reactive antibody fraction from a single *Pf*AMA1 allele immunisation may not be as efficient for achieving significant parasite inhibition.

## Discussion

Allelic polymorphism in AMA1 is due to single amino acid substitutions and has been linked with host immune pressure on the parasite [15], [25]. Although this makes AMA1 a possible target for natural as well as vaccine induced responses, polymorphism presents the practical challenge of developing a broadly effective vaccine since immunisation with one AMA1 haplotype appears not to protect against parasites expressing relatively distant haplotypes [12]. Preliminary analysis of about 745 *Pf*AMA1 amino acid sequences pulled from PubMed through GeneBank shows 236 unique AMA1 haplotypes, with an estimated 189 occurring in domain I alone (unpublished data). An effective vaccine is expected to protect against this diversity of parasites globally or at least within a particular endemic region. It is worth noting that while about 10% of amino acid residues in the AMA1 ectodomain are polymorphic, most of these residues are dimorphic, a few are tri- or tetramorphic, and a single position in domain I (197) is heptamorphic [25], [28]. Polymorphic residue linkages present within the molecule also tend to limit the choice of amino acids in certain polymorphic positions [18], providing some level of polymorphic stability. Overcoming *Pf*AMA1 polymorphism is therefore a key step in vaccine development, and immunisation with a mixture of *Pf*AMA1 alleles has been proposed as one possible solution to this challenge [3], [17], [18], [37].

The aim of the present study was to determine the relative functional importance of cross-reactive and strain-specific antibody fractions elicited upon immunisation with a particular *Pf*AMA1 allele, and also to assess the feasibility of achieving broad strain recognition by antibodies to a multi-allele *Pf*AMA1 vaccine. We validated a competition ELISA assay for assessing antibody specificities providing a highly reproducible and robust methodology for dissecting specific antibody responses to immunisation with a *Pf*AMA1-based vaccine. The assay was independent of the specific antibody source (serum or purified IgG) and the antibody dilution factor when the working OD values fell within the linear portion of the standard/calibration curve. The latter observation may be explained by the fact that the antigen-antibody complex reaction, being reversible, would always have very similar percentage proportions of reaction components when it attains a dynamic equilibrium state. It is also worthwhile noting that the anti-*Pf*AMA1 antibody depletion patterns observed for the various competitor *Pf*AMA1 alleles in competition assays were generally predictive of the extent of growth inhibition of the different parasite strains by the anti-*Pf*AMA1 antibodies *in vitro*.

The major findings of this study are that i) immunisation with a mixture of allelic *Pf*AMA1 forms predominantly induces the production of cross-reactive anti-*Pf*AMA1 antibodies, and ii) the cross-reactive fraction of antibodies to any *Pf*AMA1 allele has the same functional capacity (GIA) as the total anti-*Pf*AMA1 antibodies (cross-reactive+strain-specific) at the same concentration, hence induction of antibodies to epitopes that are common to a number of *Pf*AMA1 alleles will not reduce the inhibitory capacity against parasites expressing any of the vaccinating alleles.

The increased recognition and depletion of anti-Combi antibodies by all competitor antigens, including the out-group antigen CAMP, compared to that of mono-specific antibodies implies a broadened antibody response (Figure 1). This is most likely due to the induction of antibodies predominantly to epitopes that are common to the component alleles of the vaccine, and similar observations have been made in earlier studies [17], [27], [37]. Since strain-specific epitopes on each vaccine allele are expected to be present at relatively low quantities in the mixture, they will have a decreased probability of presentation to the relevant immune system effectors compared to common epitopes. Indeed, the levels of antibodies to common epitopes in the mixed allele immunisation, as assessed by competition ELISA assays, were constantly higher relative to the levels in antibody pools made from the three single immunisations with the same antigens (Figure 1).

This was confirmed by the growth inhibition patterns observed when all antibody fractions were tested against the FCR3, HB3, NF54 and CAMP parasite strains (Figure 2).

Recognition and depletion of affinity-purified IgG fractions from FVO and 3D7 AMA1 mono-specific immunisations provide further evidence for the induction of antibodies against common epitopes. Most (≥80%) of the cross-reactive epitopes between these two *Pf*AMA1 alleles are shared with CAMP and HB3 AMA1 alleles (Figure 3). Additionally, for the anti-3D7 IgG fraction, affinity depletion of anti-FVO cross-reactive antibodies removed up to 80% of antibodies reactive to the HB3 and CAMP AMA1 alleles. These observations may possibly extend to the many other *Pf*AMA1 alleles not tested in this study. The patterns of recognition and depletion of the affinity-purified, cross-reactive fractions closely resemble those of antibodies induced by the mixed allele immunisation in ELISA.

Observations from competition ELISA were confirmed by functional parasite growth inhibition assays with the FCR3, NF54, HB3 and CAMP strains of *P. falciparum*. The extent of antigen recognition by these antibody fractions was predictive of the degree of functional parasite inhibition observed *in vitro*. Furthermore, assays with affinity-purified anti-*Pf*AMA1 antibodies on FCR3 and NF54 parasite strains showed that at the same concentration, the cross-reactive fractions had the same growth inhibitory effects as the total anti-*Pf*AMA1 IgGs on homologous parasites, while the strain-specific fractions had slightly lower inhibitions over the IgG concentrations tested (Figure 4). Thus in the absence of the strain-specific fraction of antibodies against any *Pf*AMA1 allele, the cross-reactive fraction alone is still highly inhibitory against homologous parasites. The limited cross inhibition of heterologous parasites by antibodies from single allele immunisations may therefore be attributed to the proportions of cross-reactive and strain-specific antibodies; the cross-reactive antibody fraction may not be enough to effectively inhibit heterologous parasite invasion of RBCs to the same extent as homologous strain inhibition, which would involve both cross-reactive and strain-specific antibody activity.

Cross-reactive antibody fractions of both anti-FVO and anti-3D7 AMA1 IgGs, initially derived from mono-specific sera, inhibited the respective heterologous strains less effectively *in vitro* (Figure 4). Since the same concentration of both antibody fractions resulted in greater growth inhibition of the respective homologous parasites, the current observation may be a potential consequence of the affinity purification process, or logically due to avidity differences in binding to homologous and heterologous parasite *Pf*AMA1 alleles. This latter observation, if confirmed, would imply that the cross-reactive fraction of antibodies generated by single allele immunisation may not be as equally good as cross-reactive antibodies induced by multi-allele immunisation in terms of functional capacity against heterologous parasites.

The measured avidity indices for anti-Combi and mono-specific antibodies on the immunising antigen(s) as well as for mono-specific antibodies on “heterologous” *Pf*AMA1 alleles were comparable (Table 4). Due to antibody titre normalization however, the quantity of each mono-specific serum that was used for the avidity determination on heterologous alleles was up to 2-fold higher than the quantity of mono-specific and anti-Combi antibodies used on the respective homologous alleles. Thus cross-reactive antibodies, irrespective of the source, bound the allelic *Pf*AMA1antigens to very similar degrees, and the only factor that influences the extent of *in vitro* parasite inhibition was the absolute levels of these functional antibodies. These observations, taken together with the fact that the functional activity of antibodies is generally linked with their antigen binding strength [38], [39], support the conclusion that anti-Combi antibodies are most likely high-avidity in nature. Additionally, most low-avidity antibodies are likely to be lost during affinity purification of *Pf*AMA1-specific antibodies. Depletion patterns for the affinity-purified cross-reactive fractions from both anti-3D7 and anti-FVO AMA1 antibodies (Figure 3) were however, similar to those of anti-Combi antibodies (Figure 1) which were Protein A-purified and should therefore include any low-avidity AMA1-specific antibodies. The absence of such low-avidity, cross-reactive antibodies after affinity purification would be expected to result in antibody depletion patterns that are rather similar to those of mono-specific antibodies (Figure 1).

Considering the data presented, it may be hypothesized that apart from antibody specificity, an optimal concentration of antibodies is also necessary in order to achieve a good degree of parasite inhibition. These findings are important for two main reasons, that i) there is no loss in *in vitro* inhibitory capacity by mainly inducing antibodies to common epitopes, and ii) the antibodies thus induced will also cross-react with epitopes on other *Pf*AMA1 alleles that are similar to those on the vaccine's component alleles to which they were raised. High titres of antibodies to such common epitopes imply broadened recognition and inhibition of a wide range of parasite strains. Taken together, these results provide evidence for the induction of high levels of inhibitory antibodies to common epitopes by immunisation with a mixture of *Pf*AMA1 alleles. An added advantage of this strategy over a typical multi-antigen or multistage vaccine is the possible limitation on the number of different antigens (with very different epitopes) that can be practically included in a multistage vaccine formulation without compromising effectiveness. Such a multistage vaccine may induce low antibody titres to each of a wide variety of antigens/epitopes, some of which may not be anti-parasitic enough, such that the overall response will be affected by the reduced effective antibody concentration [27], [40], [41]. A multi-allele strategy with a promising antigen like *Pf*AMA1, by comparison, will focus the humoral response on relevant epitopes that are common to all constituent alleles. In theory, increasing the number of constituent alleles will reduce the number of common/overlapping epitopes and these repeated epitopes will form the bulk of all epitopes present in the mixture. This would be expected to translate to relatively higher antibody titres against these common epitopes, with the result being broad antibody specificity. Duan and others [42] have recently proposed such an approach, with a recommended minimum of six *Pf*AMA1 alleles as components of a universal vaccine. The number of alleles that can be practically included in such a vaccine may however be limited by high costs (of producing six different proteins) and/or practical developmental difficulties (of expressing fusion proteins with six component antigens), especially if AMA1 is to be combined with other highly immunogenic antigen(s) in a multi-antigen vaccine. On this basis, the diversity covering approach [18], comprising three synthetic and highly divergent *Pf*AMA1-based proteins, appears to be a more practical approach to *Pf*AMA1 vaccine development. Being highly divergent sequences, the three DiCo antigens are expected to have fewer common/overlapping antibody epitopes. These would nevertheless represent the greater proportion of antibody epitopes in the DiCo mixture, and would induce high antibody titres upon immunisation. It is therefore practically possible and more cost-effective to induce a significantly broad humoral response to *P. falciparum* strains using the DiCo proteins, at least as components of a multi-antigen vaccine.

In summary, the present study has shown that broad functional specificity of anti-*Pf*AMA1 antibodies to diverse *P. falciparum* strains can be achieved by multi-allele immunisation. The humoral response is most likely focused on epitopes that are common to the constituent alleles, which would form the bulk of all epitopes present, and leads to induction of antibodies to these common epitopes. The results also show that majority of B cell epitopes are shared by the *Pf*AMA1 alleles used in this study, and possibly by many other *Pf*AMA1 alleles. Thus antibodies induced against a multi-allele vaccine are also highly likely to be effective against parasites expressing diverse *Pf*AMA1 alleles. Of central importance to this immunisation strategy is the demonstration of good levels of homologous parasite inhibition by cross-reactive anti-*Pf*AMA1 antibodies, compared to the total antibody fraction at the same concentration *in vitro*. This is necessary to ensure that in aiming to broaden the antibody response, functionality against parasites expressing the vaccine *Pf*AMA1 alleles is not compromised.

## Supporting Information

Figure S1 Schematic presentation of strain-specific and cross-reactive anti-AMA1 antibody purification. Cross-reactive and strain-specific IgG fractions of anti-3D7 AMA1 IgGs (A) and anti-FVO AMA1 IgGs (B) were isolated from sera of the respective mono-specific AMA1-immunised rabbits. Serum IgGs were first purified over protein A sepharose columns before affinity fractionation.
(0.13 MB TIF)

Figure S2 Competition ELISA using different dilutions of anti-FVO AMA1 antibodies with FVO AMA1-coated plates. The assay involves co-incubation of a soluble/competitor antigen with antibodies in an antigen-coated plate such that there is competition between the coated and soluble/competitor antigens for binding to test antibodies. Protein A-purified anti-FVO AMA1 antibodies were used at dilutions equivalent to 0.2, 0.5, 1, 2, 4 and 8 times the antibody titre (1 AU, the IgG dilution that yields an OD405 of 1.0). Each dilution of antibody was added to FVO AMA1-coated plates with soluble/competitor AMA1 antigens from the 3D7, HB3, FVO and CAMP parasite strains, each titrated from 30–0.005 µg/ml in duplicate wells. Antibodies that are not depleted by the soluble/competitor antigens bind to the coated antigen (residual binding), and the resulting optical densities (OD) were expressed as percentages of ODs from reagent wells with antibody but no competitor antigens. Competitor antigen concentrations (log transformed) were then plotted against the percent residual binding for all competitor antigens. Depletion patterns for competitor/soluble FVO or sFVO (A), sHB3 (B), s3D7 (C) and sCAMP (D) AMA1 antigens at the different antibody dilutions are shown.
(0.09 MB TIF)
