# Supplementary figures and images for: Humoral Immune Response to Mixed PfAMA1 Alleles; Multivalent PfAMA1 Vaccines Induce Broad Specificity
Source: PLoS One. 2009 Dec 1;4(12):e8110. doi: 10.1371/journal.pone.0008110 (PMC2779588; doi:10.1371/journal.pone.0008110)

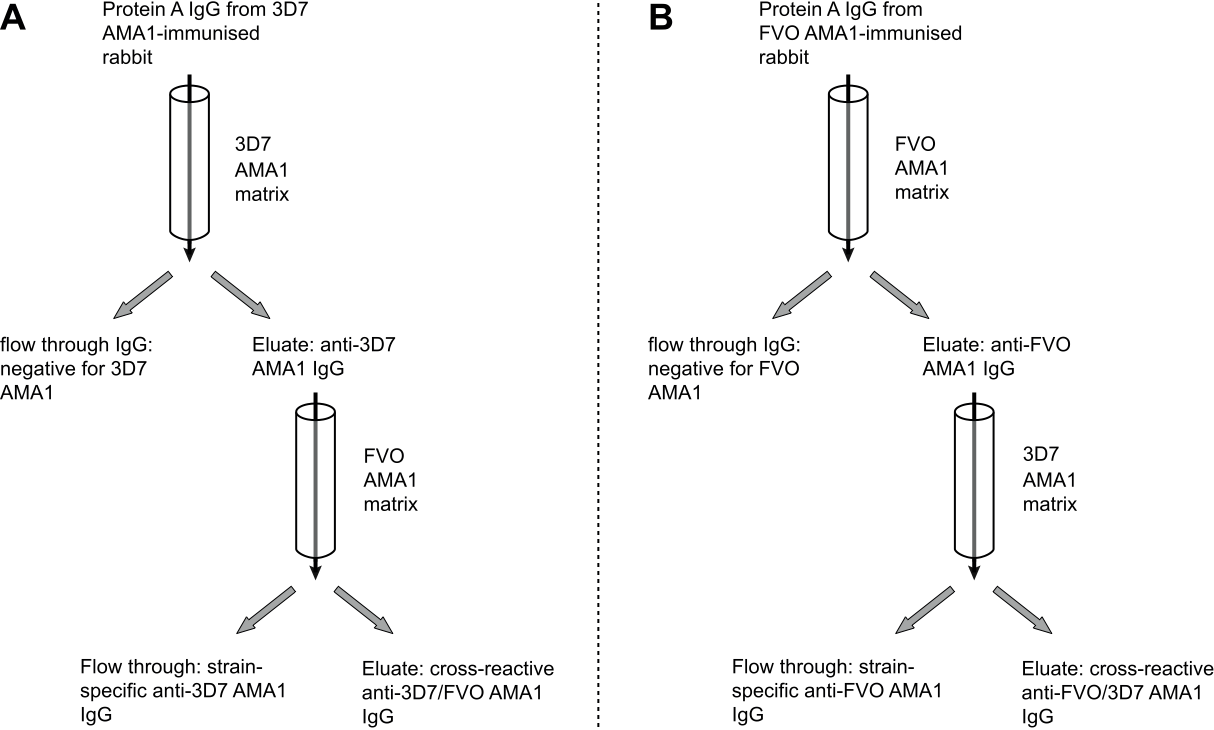

Supplement: Figure S1 — Schematic presentation of strain-specific and cross-reactive anti-AMA1 antibody purification. Cross-reactive and strain-specific IgG fractions of anti-3D7 AMA1 IgGs (A) and anti-FVO AMA1 IgGs (B) were isolated from sera of the respective mono-specific AMA1-immunised rabbits. Serum IgGs were first purified over protein A sepharose columns before affinity fractionation. (0.13 MB TIF) [file pone.0008110.s001.tif]

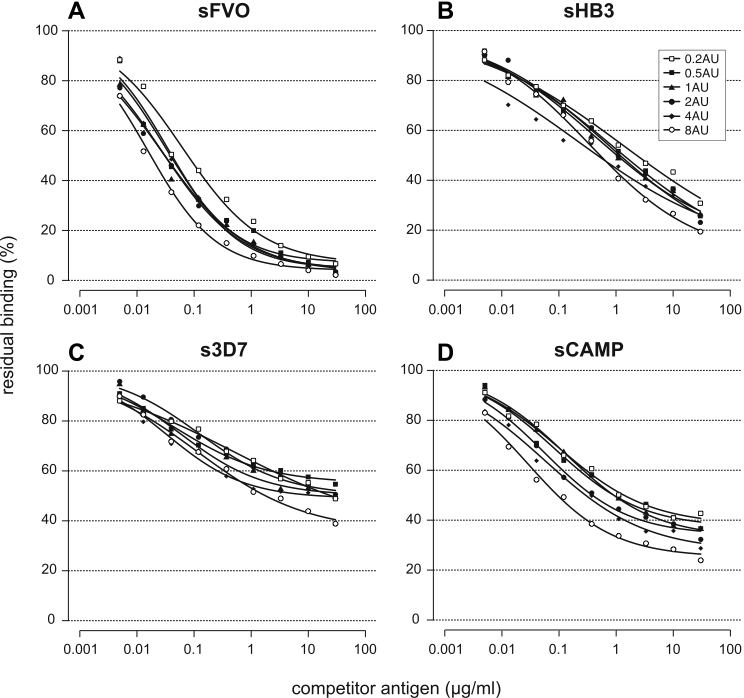

Supplement: Figure S2 — Competition ELISA using different dilutions of anti-FVO AMA1 antibodies with FVO AMA1-coated plates. The assay involves co-incubation of a soluble/competitor antigen with antibodies in an antigen-coated plate such that there is competition between the coated and soluble/competitor antigens for binding to test antibodies. Protein A-purified anti-FVO AMA1 antibodies were used at dilutions equivalent to 0.2, 0.5, 1, 2, 4 and 8 times the antibody titre (1 AU, the IgG dilution that yields an OD405 of 1.0). Each dilution of antibody was added to FVO AMA1-coated plates with soluble/competitor AMA1 antigens from the 3D7, HB3, FVO and CAMP parasite strains, each titrated from 30–0.005 µg/ml in duplicate wells. Antibodies that are not depleted by the soluble/competitor antigens bind to the coated antigen (residual binding), and the resulting optical densities (OD) were expressed as percentages of ODs from reagent wells with antibody but no competitor antigens. Competitor antigen concentrations (log transformed) were then plotted against the percent residual binding for all competitor antigens. Depletion patterns for competitor/soluble FVO or sFVO (A), sHB3 (B), s3D7 (C) and sCAMP (D) AMA1 antigens at the different antibody dilutions are shown. (0.09 MB TIF) [file pone.0008110.s002.tif]
